# Non-Photosynthetic Melainabacteria (Cyanobacteria) in Human Gut: Characteristics and Association with Health

**DOI:** 10.3390/life12040476

**Published:** 2022-03-25

**Authors:** Chenlin Hu, Piotr Rzymski

**Affiliations:** 1 College of Pharmacy, University of Houston, Houston, TX 77204, USA; 2 Department of Environmental Medicine, Poznan University of Medical Sciences, 60-806 Poznan, Poland; 3 Integrated Science Association (ISA), Universal Scientific Education and Research Network (USERN), 60-806 Poznań, Poland

**Keywords:** human microbiome, gut, non-photosynthetic cyanobacteria, Melainabacteria, human disease

## Abstract

Gut microorganisms are comprised of thousands of species and play an important role in the host’s metabolism, overall health status, and risk of disease. Recently, the discovery of non-photosynthetic cyanobacteria (class “Melainabacteria”) in the human and animal gut triggered a broad interest in studying cyanobacteria’s evolution, physiology, and ecological relevance of the Melainabacteria members. In the present paper, we review the general characteristics of Melainabacteria, their phylogeny, distribution, and ecology. The potential link between these microorganisms and human health is also discussed based on available human-microbiome studies. Their abundance tends to increase in patients with selected neurodegenerative, gastrointestinal, hepatic, metabolic, and respiratory diseases. However, the available evidence is correlative and requires further longitudinal studies. Although the research on Melainabacteria in the human gut is still in its infancy, elucidation of their role appears important in better understanding microbiome–human health interactions. Further studies aiming to identify particular gut cyanobacteria species, culture them *in vitro*, and characterize them on the molecular, biochemical, and physiological levels are encouraged.

## 1. Introduction

Cyanobacteria are the oldest organisms on earth, and their fossil record possibly tracked back to ~3.5 billion years ago [1]. The emergence of the oxygenic photosynthesis of cyanobacteria was associated with the rise of oxygen in the earth’s atmosphere (also known as the Great Oxygenation Event) ~2.1 billion years ago [2]. Over the long evolutionary life, cyanobacteria have adapted to various changing environments and present high diversity in morphology, metabolism, and eco-physiology [3]. Cyanobacteria are ubiquitous and inhabit a broad spectrum of freshwater, marine, and terrestrial habitats, including extreme environments, e.g., hot spring, desert crusts, and polar zones. Cyanobacteria constitute the important primary producers and play a critical role in the global biogeochemical cycling of carbon and nitrogen [3]. They also attract attention due to their ability to form massive blooms that deteriorate water quality and threaten public health by producing toxic metabolites representing various chemical classes [4]. Recently, the discovery of the non-photosynthetic cyanobacteria Melainabacteria in the aphotic environments (e.g., lake sediment and aquifer as well as human and animal guts) [5,6,7,8] have advanced our understanding into the breadth and complexity of cyanobacteria and are receiving the attention relating to the origin of oxygenic photosynthesis [9], redefinition of cyanobacteria [7,10,11], and also raised the new open questions regarding the ecological relevance of the non-photosynthetic cyanobacteria, e.g., Melainabacteria in these aphotic environments, particularly human gut.

This paper provides an overview of Melainabacteria as a class of the phylum Cyanobacteria and reviews the studies on these microorganisms’ presence in the human gut microbiome with a focus on their potential health relevance.

## 2. The New Class Melainabacteria of the Phylum Cyanobacteria

The cyanobacterial 16S rRNA-like sequences have been previously detected in human gut samples [12,13,14], bovine rumen [15], termite gut [16], and another animal guts [17,18], implying the presence of non-photosynthetic cyanobacteria lineage in these aphotic environments. Di Rienzi et al. (2013), for the first time, assembled the complete genomes of non-photosynthetic cyanobacterium-like from human gut and groundwater, which were assigned to a new phylum Melainabacteria (Greek nymph of dark waters), sibling to the phylum Cyanobacteria because of the <85% sequence similarity with photosynthetic cyanobacterial members [5]. Later, Soo et al. (2014) expanded the coverage of the Melainabacteria members via assembling new Melianabacteria genomes from human and koala gut and bioreactor samples; they re-designated the Melainabacteria into a class within the phylum Cyanobacteria given the robust monophyly and shared traits with photosynthetic cyanobacteria [7]. This re-classification was supported by the genome phylogeny-based taxonomy [19]. The class Melainabacteria is divided into six major taxonomic orders (*Vampirovibrionales*, *Obscuribacterales*, *Gastranaerophilales*, and *Caenarcaniphilales*, SHAS531, and V201-46) based on the habitat and analysis of population genomes [7,9], the former and middle two are proposed to the microaerophilic and obligate anaerobic members, respectively, and the latter two orders have not been defined yet [6]. Soo et al. (2015) recently identified a predatory bacterium (*Vampirovibrio chlorellavorus*, previously known as proteobacteria) of green algae *Chlorella* to be the first cultivatable representative of non-photosynthetic cyanobacterial Melainabacteria through analyzing the genomes of lyophilized archive sample [20], the new *V*. *chlorellavorus* isolates were recently sequenced and characterized [21], they present the cocci cellular shape (0.3–0.6 µm in diameter) [22]. Utami et al. (2017) utilized the single-cell sorting and sequencing technology to assemble the genome of a melainabacterium (*Candidatus Gastranaerophilus termiticola* Tpq-Mel-01) from the termite gut [6], and they further utilized the fluorescence in situ hybridization (FISH) technology to demonstrate that *G*. *termiticola* Tpq-Mel-01 grew in rod shape (1.0 µm by 0.5 µm in dimension).

So far, over 50 Melainabacteria genomes have been partially or wholly assembled from human and animal guts, bioreactor, lake water, and aquifer samples [5,6,7,9,20,21]; Table 1 summarizes the characteristics of the representative Melainabacteria members, in which the whole genome was sequenced. The *Gastranaerophilales*, *Caenarcaniphilales*, and *Vampirovibrionales* members have the relatively smaller genome size (1.6–2.7, 1.8–2.2, 2.8–3.0 Mbp, respectively) compared to the *Obscuribacterales* members (3.4–5.5 Mbp), and the *Obscuribacterales* and *Vampirovibrionales* members have relatively higher G+C content (49–55%) [7,21] compared to the other two order members (28–43%) [6,7], highlighting the genomic diversity of the Melainabacteria.

## 3. Ecology of Melainabacteria

All the Melainabacteria members are chemoheterotrophs as they lack the genes for photosynthesis and carbon fixation. Among four defined orders (*Vampirovibrionales*, *Obscuribacterales*, *Gastranaerophilales*, and *Caenarcaniphilales*), the *Gastranaerophilales* is the sole one lacking the essential genes for aerobic respiration [23]. This directly corresponds to the fact that *Gastranaerophilales* members are mainly associated with human and animal guts, which have an environment low in oxygen or nearly anoxic. *Gastranaerophilales* is predicted to acquire energy via the Embden–Meyerhof pathway that converts simple carbohydrates (e.g., glucose, mannose, and starch) into fermentation products (e.g., lactate and ethanol) [5]. Except for the ACD20 (*Gastranaerophilales*) isolated from the aquifer, all the other Melainabacteria members also lack the complete functional genetic set for nitrogen fixation [5]. Therefore, similar to photosynthetic cyanobacteria, not every Melainabacteria member is a nitrogen fixer. Four Melainabacteria members (*Gastranaerophilales*: Tpq-Mel-01, MEL_B1, MEL_B2, and ACD20) harbor the complete set of flagella genes [5,6,7]. The flagella are proposed to be present in the ancestral Melainabacteria but lost in some members during the evolution period [7]. Genomic analysis of two *Vampirovibrio chlorellavorus* isolates predicted the presence of quorum sensing and motile-related functional genes [21]. Additionally, the Melainabacteria members from the human gut harbor the genes for the biosynthesis of vitamin B (e.g., riboflavin, nicotinamide, biotin, and dihydrofolate) and vitamin K [5,7], thus they might constitute an important source of vitamin biosynthesis that is beneficial to humans.

Melainabacteria are associated with a wide spectrum of ecological niches, including the soil, water, and animal habitats [5,6,7,8,9,24]. In humans, Melainabacteria are mainly present in the gut and rarely in the respiratory tract, mouth-associated environments, or on skin surface [5]. Most of the *Gastranaerophilales* members are from human and other animal gut environment [5,7,9], while the remaining members are from the natural field environments (e.g., aquifer and lake sediment) [5,8]. The *Obscuribacterales* members have a wide inhabit range, including the soil, bioreactor, aquifer, and lake sediment [7,8,24]. In a recently conducted global survey of soil cyanobacteria, the *Obscuribacterales* members were found to dominate the Melainabacteria cluster. Moreover, they had distinctively different habitat preferences compared to the photosynthetic cyanobacteria associated mainly with humid and acidic soils in tropical and cold forests and grasslands. Therefore, as predicted, they are likely to occur most abundantly in humid areas of the Amazon Basin, Central Africa, the West Asian coast, and the Pacific Islands [24]. The reported *Caenarcaniphilales* members are from the lake water and bioreactor [7,9]. The reported *Vampirovibrionales* members belong exclusively to the parasitic bacterium *Vampirovibrio chlorellavorus* that co-occur with and predate the green algae *Chlorella*. Interestingly, the *Vampirovibrionale*-specific 16S rRNA sequences were detected in the lake sediment samples of Peri-Alpine Lakes [8]. Melainabacteria members typically occur in low abundance (~0.01–10%) in their habitats [5,6,7]. Di Rienzi et al. (2013) reported that gut Melainabacteria in the herbivore population were much more abundant than omnivore and carnivore populations, suggesting that the representatives of Melainabacteria might be involved in the digestion of dietary plant polysaccharides in humans [5]. Further dissection of the eco-physiological roles of Melainabacteria in their habitats, especially in the human and other animal gut, is warranted in the future. 

## 4. The Health Relevance of Melainabacteria in the Human Gut

Although numerous studies have addressed the relationships between human gut microbiome composition and various diseases (e.g., gastrointestinal and hepatic diseases, metabolic diseases, kidney disease, and immune-related diseases as well as mental health diseases) [25,26,27,28], the health relevance of non-photosynthetic cyanobacteria in the human gut remains yet to be studied in detail and elucidated. Culture-independent metagenomics studies allow taxonomically classifying different gut microorganisms at the levels of phylum, class, order, family, genus, and species [29]. 

Previously conducted human microbiome studies provide the opportunity to examine the potential link between gut cyanobacteria and human health and diseases. In the human gut, the predominant bacteria are Firmicutes, Bacteroidetes, Proteobacteria, Fusobacteria, Cyanobacteria, Verrucomicrobia, and Euryarchaeota [30]. If gut cyanobacteria are essentially associated with human health and disease, they would be expected to vary significantly between different subject groups (e.g., healthy group vs. certain disease-associated group). Based on this rationale, we carried out a review of such comparative human gut microbiome studies to understand the potential health relevance of gut cyanobacteria. The literature search was conducted through Web ISI and PubMed databases with the use of the combination of keywords: “cyanobacteria”, “human,” and “gut”). All retrieved papers published till the end of 2021 were considered for further assessment. The inclusion criteria for the review included: original type of article, human study, analysis of human gut microbiome, investigation of the relationship between gut cyanobacteria and human health. Overall, thirteen studies were reviewed (Table 2).

### 4.1. Neurodevelopment and Neurodegeneration

Selected cyanobacteria can synthesize a non-essential neurotoxic amino acid, β-methylamino-L-alanine (BMAA) [44], which is suggested as a potential etiological factor of neurodegenerative processes and diseases (e.g., Amyotrophic Lateral Sclerosis (ALS), Parkinson’s disease, and dementia). This raises the question of whether such BMAA-producing microorganisms can be a part of the human intestinal microflora [14]. Therefore, it has been hypothesized that the BMAA-producing cyanobacteria are present in the human gut and are associated with the development of neurodegenerative diseases (e.g., ALS, Alzheimer’s disease, and Parkinson’s disease) in humans [45]. However, no clinical studies have been performed to confirm these hypotheses. Di Gioia et al. (2020) performed the first prospective longitudinal study analyzing the compositional gut microbiota difference between 50 ALS patients and 50 control subjects matched for sex, age, geographical origin, and eating habits [31]. In their double-blinded, placebo-controlled phase I pilot trial, the patients received either probiotic supplement or placebo to assess the impact of probiotic supplementation on the gut microbiota and disease progression. Interestingly, they observed that members of the Cyanobacteria phylum in the diseased group were significantly higher than those in the control group (0.3% vs. 0.2%, respectively; *p* < 0.05).

Such a differing pattern was also observed for the Gastranaerophilales members belonging to the non-photosynthetic cyanobacterial Melainabacteria (*p*  <  0.05). Their findings supported the hypothesis of the potential role of gut cyanobacteria in the pathogenesis of neurodegenerative diseases [31,33]. More recently, a population study was conducted to investigate the impact of exposure to polycyclic aromatic hydrocarbons on neurodevelopment on the gut composition in 38 healthy three-year-old healthy children that had postnatal PAH exposure [32]. After adjusting for the urinary hydroxyl PAHs, the cyanobacteria abundance was negatively correlated with the neurodevelopment in adaptation, gross motor, and language [32]. These findings continued indirectly to support the hypothesis of the association of gut cyanobacteria and neurodevelopment disorder. Furthermore, more detailed studies are needed to evaluate it.

### 4.2. Gastrointestinal and Metabolic Diseases

When studying the association of the human gut microbiome with gastrointestinal and hepatic diseases, Lu et al. (2016) analyzed the adenoma mucosal biopsy samples and adjacent normal colonic mucosa from 31 patients with adenomas and 20 healthy controls. Significantly higher cyanobacterial abundance in the adenomatous tissue was found when compared to the healthy tissue [33]. Xiong et al. (2021) analyzed the fecal samples from 25 healthy infants in comparison to samples collected from 18 and 24 infants with acute gastroenteritis caused by rotavirus and human norovirus, respectively. Cyanobacteria members had a higher abundance in infants’ gut with viral diarrhea compared to the healthy control group [34]. These two studies consistently implied the positive correlation between gut cyanobacteria and gastrointestinal disease. Sarangi et al. (2017) analyzed the fecal samples from 35 patients with cirrhosis and 18 healthy controls; and they observed a relatively lower cyanobacterial abundance in the patients with cirrhosis compared to the health controls (0.0% vs. 0.53%, *p* < 0.05) [35], suggesting the negative correlation between the gut cyanobacteria and cirrhosis. One should note that such studies are insufficient to imply causation. Further longitudinal studies are warranted to assess it.

In terms of metabolism-associated health and disease, Kaplan et al. (2019) analyzed the gut microbiome composition in the 1674 adults of Hispanic Community Health Study/Study of Latinos in the USA, and they observed that the cyanobacteria were significantly negatively associated with obesity [36]. Oduaran et al. (2020) analyzed the population of South Africa and observed a significant abundance of the Vampirovibrio members (non-photosynthetic cyanobacterial Melainabacteria) in the rural community Bushbuckridge when compared to inhabitants the highly urbanized area Soweto [37]. Chumpitazi et al. (2019) noticed that the fructan-sensitive children with irritable bowel syndrome have enriched cyanobacteria compared to the fructan-tolerant group, indicating the involvement of cyanobacteria in food digestion [38]. Cai et al. (2020) analyzed the gut microbiota composition in the patients with Wilson’s disease (an autosomal recessive inherited disorder of chronic copper toxicosis), and they observed a higher cyanobacterial abundance in the patients with Wilson’s disease compared to the health controls (0.12% vs. 0.0%, *p* < 0.05) [39]. It appears that there is no consistently positive or negative correlation between gut cyanobacteria and metabolism-associated disease.

### 4.3. Other Diseases

Zhu et al. (2020) analyzed the gut microbiota composition in the patients with allergy rhinitis (AR) and showed that the cyanobacterial abundance in the AR patients was significantly lower than those in the non-AR group [40]. Zhang et al. (2018) observed a relatively higher gut cyanobacterial abundance in lung cancer patients than the healthy controls [41]. Sublette et al. (2020) analyzed the intestinal microbiota in the abstainers and continuing smokers and observed that the exhaled CO level in daily cigarette smokers correlated positively with the relative abundance of gut cyanobacteria [42]. These findings suggest the need for further studies elucidating the potential role of gut cyanobacteria in respiratory diseases. Shi et al. (2021) analyzed the gut microbiota composition of 30 Graves’ disease (GD) patients without Graves’ orbitopathy (GO), 33 GO subjects, and 32 healthy subjects and observed the significant difference in the gut cyanobacteria among the studied groups, implying the potential association of the changing gut cyanobacterial abundance with the Graves’ disease and Graves’ orbitopathy [43]. 

## 5. Conclusions

**Figure 1:**
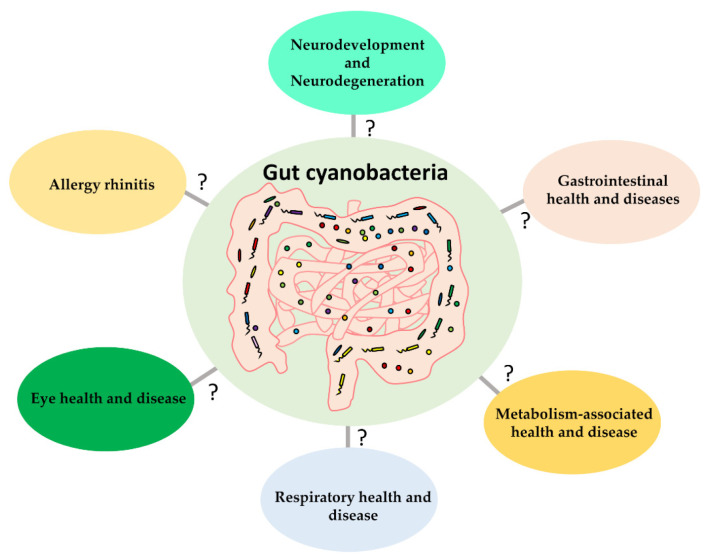
The potential link of gut cyanobacteria with human diseases and health.

At present, gut cyanobacteria research is still in its very infancy. Melainabacteria representatives are the only non-photosynthetic cyanobacterial members that were discovered in the gut environment of humans and animals. Taxonomically, Melainabacteria was assigned to a new class of cyanobacteria and is further divided into at least four taxonomic orders (*Vampirovibrionales*, *Obscuribacterales*, *Gastranaerophilales*, and *Caenarcaniphilales*). Gastranaerophilales members are closely associated with the human and animal gut environment. Sequence analysis implied that all these Melainabacteria members are chemoheterotrophs and exhibit genomic diversity. Moreover, the presence of the genes for the vitamin biosynthesis further implied that gut Melainabacteria members might be beneficial to the host. The reviewed studies indicate significant differences in the gut cyanobacterial abundance between health control and various diseased groups implying that these organisms can somehow be related to neurodevelopment, neurodegeneration, obesity, allergy rhinitis, and gastrointestinal, respiratory, and eye diseases (Figure 1).

The available studies provide first insights into the association between these microorganisms and selected diseases, but the evidence is only correlative. It remains to be elucidated whether gut cyanobacteria can contribute to any particular disease or whether their abundance changes result from the disorder. Further research aiming to identify particular gut cyanobacteria species, culture them *in vitro*, and characterize them on the molecular, biochemical, and physiological levels is encouraged to understand these microorganisms’ role in human health and disease development.

## Figures and Tables

**Table 1:** Characteristics of the representative Melainabacteria members for which the whole genome was sequenced.

Strain	Environment	Genome Size (Mbp)	Completeness	GC (%)	Anaerobic	Flagella	Shape	Reference
Order Gastranaerophilales								
**ACD20**	aquifer	3.0	Near Complete	33.5	anaerobic	+	N.A.	[5]
**MEL.A1**	human gut	1.9	Complete	32.9	anaerobic	−	N.A.	[5]
**MEL.A2**	human gut	1.2	Partial	30.6	anaerobic	−	N.A.	[5]
**MEL.B1**	human gut	2.3	Complete	35.3	anaerobic	+	N.A.	[5]
**MEL.B2**	human gut	2.3	Complete	36.3	anaerobic	+	N.A.	[5]
**MEL.C1**	human gut	2.1	Complete	34.1	anaerobic	−	N.A.	[5]
**MEL.C2**	human gut	2.2	Near Complete	35.3	anaerobic	−	N.A.	[5]
**MEL.C3**	human gut	1.3	Partial	29.9	anaerobic	−	N.A.	[5]
**Tpq-Mel-01**	termite gut	0.96	Partial	42.5	anaerobic	+	Rod	[6]
**Zag_221**	koala gut	1.8	complete	38.5	anaerobic	−	N.A.	[7]
**Zag_1**	koala gut	2 ^a^	Near Complete	34.9	anaerobic	−	N.A.	[7]
**Zag_111**	koala gut	2.2 ^a^	Near Complete	36.7	anaerobic	−	N.A.	[7]
**MH_37**	human gut	2.2	complete	34.1	anaerobic	−	N.A.	[7]
**Order Caenarcaniphilales**								
**UASB_169**	bioreactor	1.8	complete	27.5	anaerobic	−	N.A.	[7]
**Order Obscuribacterales**								
**EBPR_351**	bioreactor	5.5	near complete	49.4	microaerophilic	−	N.A.	[7]
**Order Vampirovibrionales**								
**NCIB 11383**	coculture with *Chlorella vulgaris*	3.0	complete	51.4	microaerophilic	+	N.A.	[20]
**AZ_1**	algal cultivation ponds	2.8	near complete	54.8	microaerophilic	+	Sphere	[21,22]
**AZ_2**	algal cultivation ponds	3.0	near complete	53.0	microaerophilic	+	Sphere	[21,22]

^a^—Predicted genome size that was calculated according to the completeness of the whole genome sequencing; N.A.—not available.

**Table 2:** Summary of the potential association of gut cyanobacterial abundance with human health and diseases.

Country	Year	Subject	Disease	Gut Cyanobacterial Abundance	Remark	Reference
**Italy**	2020	Control group (*n* = 50) *vs*. diseased group (*n* = 50)	Amyotrophic lateral sclerosis	Disease group > control group	The finding indicated that cyanobacteria could be involved in the pathogenesis of neurodegenerative diseases	[31]
**China**	2021	3-year old healthy children (*n* = 38)	Neurodevelopment disorder	Gut cyanobacteria negatively correlated with the neurodevelopment in Adaptation	The finding implied the negative effect of cyanobacteria on neurodevelopment in adaptation	[32]
**China**	2016	Control group (*n* = 20) *vs*. diseased group (*n* = 31)	Colon adenomas	Disease group > control	The finding implied the association between the colorectal pre-neoplastic lesion and the increase in gut cyanobacterial abundance.	[33]
**China**	2021	Healthy infants (*n* = 25), infants with acute rotaviral gastroenteritis (*n* = 18), infants with acute noraviral gastroenteritis (*n* = 24)	Acute gastroenteritis	Disease group > control	The finding implied the association between human norovirus infection and the increase in gut cyanobacteria.	[34]
**India**	2017	Control group (*n* = 18) *vs*. diseased group (*n* = 35)	Cirrhosis	Disease group < control	The finding implied the association between cirrhosis and the reduction in gut cyanobacteria.	[35]
**USA**	2019	Hispanic/Latino adults (n = 1647)	Obesity	Gut cyanobacteria negatively correlated with obesity	The finding implied the negative association between obesity and gut cyanobacteria.	[36]
**South** **Africa**	2020	Rural community (*n* = 119) *vs*. urban community (*n* = 51)	-	Rural community > urban community	Gut Melainabacteria can be more abundant in the rural populations	[37]
**USA**	2020	Frutcan-sensitive (*n* = 17) *vs*. frutcan-tolerant children *(n* = 21) with irritable bowel syndrome	Irritable bowel syndrome	Fructan-sensitive > fructan-tolerant	Fructan-sensitive children were enriched in the gut cyanobacteria during fructan challenge.	[38]
**China**	2020	Control group (*n* = 16) *vs*. diseased group (*n* = 14)	Wilson’s Disease	Disease > Control	The finding implied the association between the Wilson’s disease and the higher gut cyanobacterial abundance.	[39]
**China**	2020	Control group (*n* = 31) *vs*. allergy-rhinitis (AR) group (*n* = 33)	Allergy Rhinitis	Disease > Control	The finding implied the association between allergy rhinitis and the higher gut cyanobacterial abundance.	[40]
**China**	2018	Healthy controls (*n* = 41) vs. Lung cancer (*n* = 41)	Lung cancer	Disease group > Control	The lung cancer group had a significantly higher level of gut cyanobacteria compared to the health group.	[41]
**USA**	2020	Smokers (*n* = 36)	-	Gut cyanobacteria positively correlated with exhaled CO levels	The finding implied the positive association between CO level and gut cyanobacteria among the current smokers.	[42]
**China**	2021	Patients with Graves’ disease (*n* = 30), Graves’ orbitopathy (*n* = 33) vs. healthy subjects (*n* = 32)	Graves’ disease Graves’ orbitopathy	Significant difference in the gut cyanobacterial abundance among the studied groups.	The finding implied the association of the changing gut cyanobacterial abundance with Graves’ disease and Graves’ orbitopathy.	[43]

## Data Availability

Not applicable.
